# Colectomy rate in steroid-refractory colitis initially responsive to cyclosporin: a long-term retrospective cohort study

**DOI:** 10.1186/1471-230X-7-13

**Published:** 2007-03-27

**Authors:** Giovanni C Actis, Maurizio Fadda, Ezio David, Anna Sapino

**Affiliations:** 1 Department of Digestive Disease and Nutrition; Ospedale Molinette; Torino 10126, Italy; 2 Department of Biomedical Science and Human Oncology, University of Torino, Ospedale Molinette, Torino 10126, Italy

## Abstract

**Background:**

There is consistent evidence that 50% of patients with acute, steroid-resistant flare of ulcerative colitis (UC) may achieve remission and avoid colectomy if treated with cyclosporin (CsA). However, follow-up of the responders has shown that most of them relapse and need surgery shortly after the response. We compared the records of our CsA-treated patients with those of other groups in order to help clarify this matter.

**Methods:**

All patients admitted consecutively to our Unit with an attack of UC and treated with CsA between January 1991 and December 1999 were studied. Patients were begun on continuously-infused CsA at 2 mg/kg/day (1991–1996), or on NEORAL at an initial dose of 5 mg/kg/day (1996–1999). The maintenance treatment included oral CsA for 3–6 months with or without azathioprine (AZA). CsA failure was defined as a relapse requiring steroids with or without progression to colectomy; the cumulative probability of relapse/colectomy was assessed by Fisher's exact tests and Kaplan-Meier analysis.

**Results:**

Among the patients, 39/61 (63%) initially responded. These 39 included a fatality and 4 drop-outs (unrelated to the side-effects of CsA), leaving 34 patients for the study. Of these, 61% and 35% were colectomy-free at 1 and 7 years, respectively; the corresponding figures were 80 and 60% respectively in the subset treated with AZA, but 47% and 15% in the AZA-untreated subgroup (p= 0.0007 at 7 years). Among the 34 patients, 44% were relapse-free at 1 year, but all had relapsed at 7 years (p = 0.0635). The overall resort to colectomy was 72%, while 19% of the patients remained colectomy-free.

**Conclusion:**

Sixty percent of a cohort of patients with steroid-refractory colitis responded to CsA and 60% of these responders retained the colon after 1 year. These figures fell to 35% at 7 years but improved to 60% on AZA. The overall need for colectomy remains high in these patients and toxicity must be monitored.

## Background

On the basis of the positive experience achieved in controlling organ rejection in the previous decade, the use of CsA to control acute steroid-refractory flares of UC was begun in the 1990s [1], when steroid unresponsiveness constituted a mandatory indication for colectomy in ~40% of the affected patients [2]. A controlled, albeit small, study soon showed that CsA can eliminate the need for immediate colectomy in this context [3], but the long-term efficacy of the drug in the treatment of UC has not been clearly established and remains a controversial issue. Fifteen years after the introduction of CsA to treat refractory colitis, several authors reviewed their follow-up data in an attempt to clarify the uncertainties concerning its long-term effects [4-8]. This study represents a comparative analysis between our results in treating acute steroid-refractory flares with CsA and the results obtained by others.

## Methods

Sixty-one patients formed the cohort for this retrospective study. The Institutional Advisory Board (Comitato Etico dell'Ospedale San Giovanni Battista della Città di Torino) approved our intention to extract the data for the study. Table 1 summarizes the patients' demographics, clinical features and previous treatments. Between January 1991 and April 1996, a subset of 38 patients were continuously infused with CsA through a central venous catheter for a maximum of 14 days; the initial infusion rate of 2 mg/kg/day was adjusted to maintain the whole blood CsA concentration within the upper part of the 60–240 ng/ml window established during our original kidney transplant program [9]. Between April 1996 and December 1999, the second subset of 23 patients received 5 mg/kg/day of the oral microemulsion formulation NEORAL^® ^for 3 months, with the same window as guidance. The indication for intravenous (IV) CsA in the first subset was the persistence of severe disease (13 ± 3 marks of the Rachmilewitz score [10]) after at least 1 week of parenteral steroids at 1 mg/kg.

The average daily stool passage in the Neoral subset was 7 ± 3, the first hour sedimentation rate was 53 ± 25, and the hemoglobin concentration was 10 ± 2, yielding a CAI score of 11 ± 3. As previously shown [11], the frequency of left-sided disease was the same as that in the IV treated subset.

Of the initial responders, those responding to Neoral were immediately placed on maintenance regimes; those responding to the induction phase with IV CsA were treated similarly after a 6-month phase of 6–8 mg/kg of conventional (oral) CsA. The maintenance regimes consisted of therapeutic doses of mesalamine or mesalamine derivatives, except for 4 cases, of whom one developed a reduced platelet count when receiving mesalamine. A subset of 15 responders also received AZA at an initial dose of 2 mg/kg, either concurrently with CsA (11 patients) or at the end of the course (4 cases), as detailed in the Results section.

Responses and toxicity were monitored as previously described [11,12]. By the initial response, we intended to avoid immediate colectomy. Cyclosporin failure was defined as disease relapse requiring steroids, with or without progression to colectomy.

### Statistical analysis

Data were first analyzed by descriptive statistics. Differences between groups were tested by Student's t-test, chi-square and Fisher's exact test, as appropriate. The cumulative probability and recurrence of relapse/colectomy was calculated using the actuarial life table analyses of Kaplan-Meier, using the log-rank test to compare the curves. Significance was assigned if P < 0.05. All the statistical analyses were performed using StatsDirect statistical software Version 2.5.7.

## Results

Sixty-one patients were treated with CsA following an acute episode of steroid-refractory UC: of these, 39 (63%) initially responded to CsA. All but 3 patients achieved the target CsA concentrations; a patient in the IV subset required her infusion rate to be halved owing to the concurrent use of a synergistic macrolide antibiotic; the Neoral patients included one high and one poor Neoral absorber, and the doses for these two patients were halved or doubled, respectively. The response rate within the NEORAL-treated subset was 69%. The onset of response was usually obvious within a week in both subsets and manifested itself as a reduction in rectal bleeding and a 50% decrease of the entry score. No factor that we could identify distinguished statistically between the initial responders and the non-responders (Table 2).

Among the 39 responders, there was one fatality and 4 patients dropped out. The fatality was due to autopsy-proven pulmonary embolism, which was judged not to be causally linked to CsA, and the four patients who dropped out did so for reasons other than CsA toxicity. Among the remaining 34 responders (Table 3), the overall colectomy-free rate after 1 year was 61%, 38% after 3 years and 35% after 7 years. However, the colectomy-free rates dropped to 47%, 15% and 15%, respectively, at the same time points in patients without AZA. In AZA-treated patients, the surgery-free rate was 66% after 3 years and 60% after 7, a statistically significant difference with respect to the AZA-untreated group (3 years: *p *= 0.0024; 7 years: *p *= 0.0007). Of the initial population of 61 patients, 10 subjects (16%) received de-novo AZA; in this subset the median time to relapse was 26 months and 30% had colectomy. In a second subset of 7 patients (11%), 5 were already on AZA prior to treatment with CsA and 2 had received a single course of AZA prior to treatment with CsA. In this subset, the median time to relapse was 8 months and 57% underwent colectomy; however, the small number of these AZA-pretreated patients did not allow the data to be analyzed statistically.

**Figure 1:**
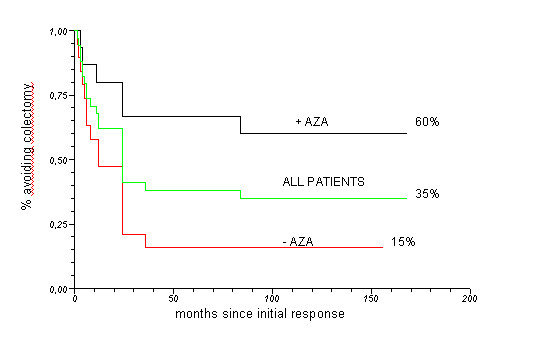
Kaplan-Meier curves of the probability of colectomy in patients taking AZA versus no AZA after induction of remission by CsA

**Figure 2:**
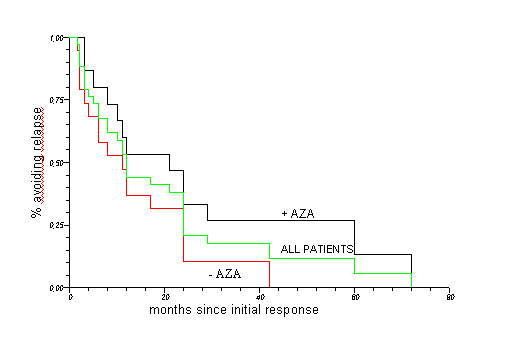
Kaplan-Meier curves of the probability of relapse in patients taking AZA versus no AZA after induction of remission by CsA.

By Kaplan-Meier analysis, the two curves describing the AZA-treated and AZA-untreated subsets were time-dependent (Fig. 1), with the hazard ratio increasing abruptly from the second year of the follow-up. The cumulative probability of colectomy differed significantly between the two curves (p = 0.007). Overall, 44% of the 34 responders were relapse-free after 1 year; however, after 7 years, all had relapsed. After 1 year, the relapse-free rate was 36% in the AZA-untreated patients, compared with 53% in the AZA-treated patients; after 3 years, the rates were 10% (- AZA) *versus *26% (+ AZA) (*p *= 0.0635) (Fig. 2). Throughout the CsA course, attempts were made to reduce the initial steroid doses: of the 30 responders with available data, 22 were on less than 10 mg of steroids daily at the end. By the end of the observation period, 72% of the cohort had undergone surgery for UC whereas 19% remained colectomy-free.

The frequencies of serious adverse effects are reported in Table 4. Tremors, hypertrichosis, and gingival hyperplasia occurred in 40–60% of patients, mainly in the oral administration phases.

## Discussion

In this retrospective study, we analyzed the immediate response and the outcome of rescue therapy with CsA in a cohort of patients whom we treated between 1991 and 1999. The patients included in this study had failed to respond to a course of full-dose parenteral steroids, and were therefore diagnosed with refractory UC, with the attendant threat of colon surgery. The findings are consistent with those from the sources quoted in the Background and indicate that: (i) the administration of IV or oral CsA can eliminate the need for immediate colectomy in 60–80% of patients with steroid-refractory UC; (ii) most of the responders will relapse with or without progression to colectomy in the following 3–7 years; (iii) the cumulative risk for colectomy in initial responders can be significantly reduced by the use of AZA as a long-acting maintenance drug. Only the report of Moskowitz is not clearly consistent with the last point. These authors did not formally conduct a comparative Kaplan-Meier analysis of the risk for colectomy in the AZA-treated versus the AZA-untreated subjects in their cohort. Rather, they claimed that de-novo treated patients were at lower risk for colectomy than those already treated with AZA at the time of the initial CsA course. The data in our paper are in fact similar to Moskowitz's findings (see the Results) and we can probably concur with them in suggesting that the AZA-pretreated subjects exhibit the poorest response to CsA in this context. However, the proportion of such patients in our study was only 11% (31% in Moskowitz's paper), which precluded stratification by Kaplan-Meier analysis. This low percentage of "bad responder" patients may be reflected in our achievement of a higher proportion of colectomy-free patients at the 7^th ^year of follow-up (35% versus 12%).

It is intriguing that while specialized UC centers like our own tend to focus on the negative side of their results, primary care centers highlight the availability of CsA for their patients and emphasize its effectiveness [13]. The data may indeed have a positive aspect: (i) if tempted to consider the action of CsA as limited or short-lived, one should take into account that all the patients enrolled in the CsA studies were by definition heading for colectomy; (ii) the often-emphasized toxicity of CsA can be controlled when the drug is administered against the background of an organ-transplant program, and can be further decreased by low-dosage regimens. The use of low-dose CsA to treat refractory colitis was originally proposed by us [14] in an uncontrolled study and later validated independently by other authors [7]. Finally, a recent controlled study has shown that low-dose CsA is as effective as high-dose [15]. Low-dose treatment might obviously impact on the toxicity figures. The rates observed in this work, although not negligible, are lower than those recorded in other high-dose trials, where for example infection and renal damage peaked to 20% [16]. The desirability of low-dose schedules has been similarly emphasized in recently published therapeutic guidelines [17], which also suggest that better patient selection and the use of purine analogues can improve the pharmacological profile of CsA in its indication for UC.

The novel biological drugs that have been introduced recently to treat UC have addressed moderately severe forms of colitis [18,19] and it is felt that their indication for treating the most severe forms of the disease is premature [17]. Also, two controlled studies published independently have come to opposite conclusions [20,21]; it may also be noted that in the study with positive findings, by Järnerot, the patients who fared worse were those with steroid-refractory disease. Doubts about the effectiveness of anti-TNF strategies in patients hospitalized with steroid-refractory disease have also been expressed in a recently published analysis [22].

## Conclusion

For patients with severe steroid-refractory UC, a strategy based on the use of CsA as an inductive therapy is effective; the progressive occurrence of relapses (eventually followed by colectomy) that follows the initial response to CsA can be significantly reduced (but not abolished) by the long-term use of a thiopurine drug (AZA); patients already on AZA will benefit least from this strategy.

## Abbreviations

ASA = Aminosalicylic acid

AZA = Azathioprine

CAI = Rachmilewitz score

CsA = Cyclosporine

IV = Intravenous

TNF = Tumor Necrosis Factor

TPN = Total Parenteral Nutrition

UC = Ulcerative Colitis

## Competing interests

The author(s) declare that they have no competing interests.

## Authors' contributions

GCA designed the study, extracted the data, and drafted the paper

MF provided the statistical basis of the work and responded to the arguments of the referee

ED and AS validated the pathological diagnoses and read and corrected the final version.

All authors read and approved the final manuscript

## Pre-publication history

The pre-publication history for this paper can be accessed here:



## Figures and Tables

**Table 1:** Patients' baseline characteristics

Mean Age (yrs)	34 ± 12
M/F	36/25
Disease Duration (months)	47 ± 7
% disease beyond splenic flexure	63
% Previous ASA use	96
% Previous AZA use	11
% Previous steroid use	100

**Table 2:** Factors of immediate response to CsA or immediate failure*

	**Immediate failure**	**Immediate Response**
Patients' age at onset (yrs)	31 ± 9	28 ± 11
Age at inclusion (yrs)	36 ± 10	34 ± 12
Disease Duration (months)	57.9 ± 13	51 ± 6
% universal disease	36	28
% transfusion dependent	36	36
% TPN dependent	52	33
White Blood Count (per μλ)	13.9 ± 7.9 × 10^3^	13.0 ± 4.8 × 10^3^
CsA levels (ng/ml whole blood)	251 ± 94	210 ± 37

* None of the differences was statistically significant

**Table 3:** Overview of the results achieved in the current cohort study.

	**All Patients **(n = 34)	**With AZA **(n = 15)	**Without AZA **(n = 19)
No colectomy > 1 yr	61^a^	80	47
No colectomy > 3 yrs	38	66	15
No colectomy > 7 yrs	35	60	15
No relapse > 1 yr	44	53	36
No relapse > 3 yrs	17	26	10
No relapse >7 yrs	0	0	0

^a ^Results are expressed as percentages

**Table 4:** Major side effects observed during the intravenous and oral phases of CsA administration

**Adverse event**	**Outcome**	**N (%)**	**Treatment Phase**
Pulmonary Embolism	Death	1 (2.5%)	Intravenous
>33% ↑ creatinine	Dose maintained	7 (17%)	Oral
2 × creatinine	Dose reduced	1 (2.5%)	Oral
Biliary sludge	Drug discontinued	2 (5%)	Intravenous
↑ liver function tests	Dose maintained	2 (5%)	Intravenous
Seizures	Dose maintained	1 (2.5%)	Oral
